# Comparative Analysis of Anodized TiO_2_ Nanotubes and Hydrothermally Synthesized TiO_2_ Nanotubes: Morphological, Structural, and Photoelectrochemical Properties

**DOI:** 10.3390/ma17215182

**Published:** 2024-10-24

**Authors:** Syrine Sassi, Amal Bouich, Brahim Bessais, Lotfi Khezami, Bernabé Mari Soucase, Anouar Hajjaji

**Affiliations:** 1 Laboratoire de Photovoltaïque, Centre de Recherches et des Technologies de l’Energie, Technopole de Borj Cédria, BP 95, Hammam-Lif 2050, Tunisia; sassi.syrine13@gmail.com (S.S.); brahim.bessais@crten.rnrt.tn (B.B.); physicshajjaji@gmail.com (A.H.); 2 Departamento de Fisica Aplicada, School of Design Engineering, Universitat Politecnica de Valencia, Cami de Vera s/n, 46022 València, Spain; bmari@fis.upv.es; 3 Department of Chemistry, College of Sciences, Imam Mohammad Ibn Saud Islamic University (IMSIU), P.O. Box 5701, Riyadh 11432, Saudi Arabia; lhmkhezami@imamu.edu.sa

**Keywords:** TiO_2_, nanotubes, hydrothermal, morphological, structural, photoelectrochemical properties

## Abstract

This study presents a comparative analysis of anodization and hydrothermal techniques for synthesizing TiO_2_ nanotubes directly on titanium foil. It emphasizes its advantages as a substrate due to its superior conductivity and efficient charge transfer. Optimized synthesis conditions enable a thorough evaluation of the resulting nanotubes’ morphology, structure, and optical properties, ultimately assessing their photoelectrochemical and photocatalytic performances. Scanning electron microscopy (SEM) reveals differences in tube diameter and organization. An X-ray diffraction (XRD) analysis shows a dominant anatase (101) crystal phase in both methods, with the hydrothermally synthesized nanotubes exhibiting a biphase structure after annealing at 500 °C. UV–Vis and photoluminescence analyses indicate slight variations in band gaps (around 0.02 eV) and recombination rates. The anodized TiO_2_ nanotubes, exhibiting superior hydrophilicity and order, demonstrate significantly enhanced photocatalytic degradation of a model pollutant, amido black (80 vs. 78%), and achieve a 0.1% higher photoconversion efficiency compared to the hydrothermally synthesized tubes. This study underscores the potential advantages of the anodization method for photocatalytic applications, particularly by demonstrating the efficacy of direct TiO_2_ nanotube growth on titanium foil for efficient photocatalysis.

## 1. Introduction

Over the past century, metal oxide materials have gained significant prominence in the scientific vocation, particularly in environmental remediation and energy resources. Their applications span diverse sectors, including solar cells, water splitting, degradation of organic pollutants and volatile organic chemicals (VOCs), electrochemical sensing, and biosensing. Among these materials, titanium dioxide (TiO_2_) has attracted considerable attention due to its beneficial properties, such as the alignment of its valence and conduction bands relative to its organic redox potential [1,2]. The increase in research on TiO_2_ underscores its effectiveness across emerging fields, particularly in environmental and energy applications, where its impact is profound [3]. In recent years, photocatalysis has been considered a possible substitute treatment for water purification. This heterogeneous approach has several benefits over conventional wastewater treatment methods and has been successfully used as an effective instrument for the degradation of a variety of dangerous chemicals, including air and aquatic organic pollutants. For example, utilizing active photocatalysts, organic pollutants can be completely degraded at room temperature in a matter of hours. Furthermore, organic contaminants can completely mineralize into comparatively non-toxic products like water and CO_2_ without producing any more dangerous byproducts [4]. The first PEC water splitting device employing TiO_2_ was exhibited by Fujishima and Honda in 2012, and since then, there have been a considerable number of related articles annually [5]. The photocatalysts in a perfect photoconversion system should have the following characteristics: (1) a high absorption coefficient and suitable band gap to maximize solar spectrum harvesting; (2) appropriate surface modification and band alignment to satisfy the kinetic and thermodynamic requirements for targeted redox reactions; and (3) high chemical robustness and photostability to allow long-term operation [6].

It has been demonstrated that altering the nanostructure and morphology of TiO_2_ can significantly affect its properties, enhancing performance if it increases the surface area or electronic mobility or diminishing it if it leads to faster recombination rates due to quick diffusion of electron–hole pairs to the surface [7,8]. Today, nanostructured TiO_2_ is found in various forms, including nanoparticles, nanobelts, nanowires, nanorods, nanotubes, and nanoflowers [9,10]. Notably, 1D nanostructures like nanotubes are advantageous for their extended surface-to-volume ratio, size confinement, atomically curved surfaces, and improved pathways for charge separation, transport, and collection, enhancing photocatalytic applications. This intricate relationship between nanostructure, morphology, and properties adds a layer of complexity to our research, making it a fascinating and challenging field to explore [11].

TiO_2_ nanotubes are extensively studied due to their significantly enlarged surface area, which increases the number of catalytically active sites, improves light harvesting, facilitates electrical transport, and minimizes the negative impacts of short diffusion lengths and recombination losses [12,13]. Various methods have been employed to synthesize TiO_2_ nanotubes, such as templating, sol–gel, anodization, and hydrothermal methods, each evolving and impacting the morphology and structure—and thus the properties—of the resultant nanotubes [14,15]. For instance, Trabelsi et al. explored how variations in anodization time affect the morphology and crystalline structure of TiO_2_ nanotubes, discovering that tubes anodized for 60 and 120 min showed the highest bacterial inactivation efficiency, attributed to optimal lengths that facilitate light penetration and reactive oxygen species (ROS) migration [16]. Similarly, Jin Yang et al. manipulated the precursor concentration and reaction temperature in hydrothermal synthesis to find conditions that yield superior performance, identifying specific samples that showed an 80% degradation efficiency of 20 mg/L methylene orange, outperforming other configurations due to differences in crystalline structure, morphology, length, density, and array ordering [17]. As the applications are diverse, each method presents TiO_2_-NTs with some superior properties that could benefit each application the most. Each method exhibits some advantages and disadvantages. Anodic oxidation is more desirable for practical applications and presents an ordered alignment with a high length-to-diameter ratio and feasibility for extensive applications, where its limitations are mass production and the difficult separation of the TiO_2_ array film from substrates. In comparison, hydrothermal synthesis is considered a simple route to obtain nanotube morphology for large-scale production, which is an advantage, and several modifications can be used to enhance the attributes of TiO_2_-NTs with a high cation-exchange capacity and length-to-diameter ratio. The disadvantages that this method presents are the difficulty in achieving uniform size, thermal instability, the need for a highly concentrated NaOH to be added, and the need for a long reaction duration [18]. 

This study aims to bridge existing knowledge gaps by synthesizing TiO_2_ nanotubes directly on titanium foil using the conventional anodization method and the modern hydrothermal approach with optimized parameters. The synthesized samples are extensively characterized morphologically, structurally, and optically to evaluate their efficacy in the photocatalytic degradation of amido black dye and their photoelectrochemical properties. This comparative analysis seeks to uncover more profound insights into how morphological and structural variations in TiO_2_ nanotubes correlate with their performance in environmental and energy applications.

## 2. Materials and Method 

### 2.1. Synthesis of Anodized TiO_2_-NTs

Titanium foils (99% purity) were sequentially polished with abrasive paper from 320 to 2000 µm grit sizes, followed by ultrasonic cleaning in acetone, ethanol, and deionized water, and then air-dried. The electrochemical anodization was performed by immersing two electrodes, spaced 2.5 cm apart, in an electrolytic bath containing 100 mL of ethylene glycol, 2 vol.% ultrapure water, and 1 vol.% ammonium fluoride (0.07 M). The titanium foil served as the anode and platinum (Pt) foil as the cathode. Anodization was conducted at 60 V for 2 h at 25 °C. The resulting TiO_2_ nanotubes were annealed at 400 °C for 3 h with a ramp of 5 °C/min to induce phase crystallization and homogenization.

### 2.2. Synthesis of Hydrothermally Grown TiO_2_-NTs Networks

The titanium foil was cleaned using ultrasonication in acetone, anhydrous ethanol, and deionized water to remove oxides and residual contaminants. The clean foil and 60 mL of 10 mol/L NaOH were placed in a polytetrafluoroethylene liner within an autoclave and then heated to 170 °C for 4 h. After cooling to room temperature, the titanium foil was immersed in 0.1 mol/L HCl for 12 h for pickling, followed by drying in a vacuum oven at 50 °C. The foils were then annealed in a muffle furnace at 500 °C for 2 h.

### 2.3. Characterization

Morphology and elemental composition were assessed using a scanning electron microscope (SEM, FEI XL30 ESEM, Hillsboro, OR, USA) and a transmission electron microscope (TEM, FEI Tecnai G2) at 200 kV with energy-dispersive X-ray spectroscopy (EDXS). X-ray diffraction (XRD) verified phase formation and stability using a Philips X’PERT-MPD diffractometer with CuKα radiation (λ = 1.5406 Å). Optical properties were investigated via UV–Vis–IR and photoluminescence (PL) spectroscopies using a PerkinElmer Lambda 950 spectrophotometer and a PerkinElmer LS55 (Waltham, MA, USA) equipped with a xenon lamp at an excitation wavelength of 340 nm, respectively.

### 2.4. Photocatalytic Process

Amido black dye (0.5 M) was used as a model pollutant. Approximately 10 mL of dye solution was placed in Petri dishes and left in darkness for 15 min to reach adsorption–desorption equilibrium. Photocatalytic degradation was tested under a germicidal OSRAM UV lamp (15 W, 45 cm, 256 nm, Wilmington, MA, USA) over 5 to 270 min intervals. Degradation efficiency was calculated using the following equation [19]:

(1) $$\eta =\left(1-\frac{A}{A_{0}}\right)100\%$$

### 2.5. Quenching of Reactive Oxygen Species

To identify the reactive oxygen species (ROS) responsible for photodegradation, specific scavengers were used: isopropanol for hydroxyl radicals (^•^OH), benzoquinone for superoxide radicals (O_2_^•−^), and ethylenediaminetetraacetic acid (EDTA) as a hole scavenger (h^+^). Each scavenger was added to the dye solution before exposure [20].

### 2.6. Photoelectrochemical Process 

Photoelectrochemical experiments were performed in a three-electrode cell comprising TiO_2_-NTs as the working electrode, Ag/AgCl in 3 M KCl as the reference electrode, and Pt wire as the counter electrode, all immersed in 1 M NaOH (pH = 13.5). A 150 W Xenon lamp provided illumination with an AM 1.5 G filter. During linear sweep voltammetry tests, the photoanodes were irradiated. The incident photon-to-current conversion efficiency (IPCE) was calculated as follows [16]:

(2) $$IPCE\left(\%\right)=\left(\frac{j_{p[E_{rev}{-|E}_{app}|]}}{I_{0}}\right)100\%$$

where *j_p_* is the current density, *E_app_ = E_mes_ − E_OC_*, Emes is the measured potential, *E_OC_* is the open circuit potential, *E_rev_* is the standard reversible potential, 1.23 V, and *I_0_* is the power of incident illumination.

## 3. Result and Interpretation

### 3.1. Morphological Characterization

**Figure 1:**
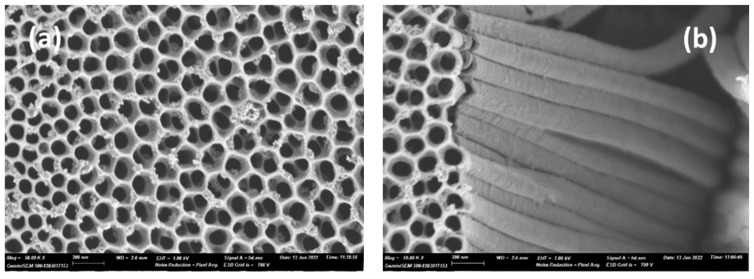

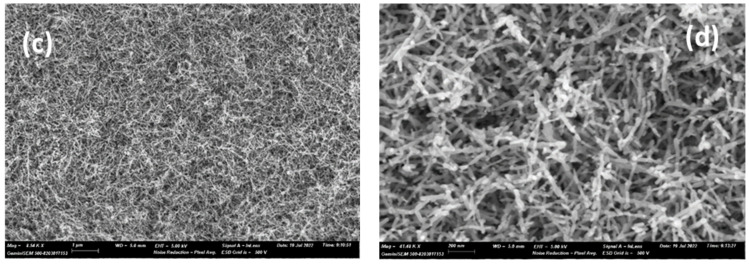
SEM images of (**a**) TiO_2_-NTs synthesized with anodization method (**b**) a close-up side view of anodized TiO_2_-NTs (**c**) TiO_2_-NT network synthesized with hydrothermal method and (**d**) a close-up view of hydrothermal TiO_2_-NTs.

The scanning electron microscopy (SEM) analysis unveiled intriguing and novel morphological differences between the TiO_2_ nanotubes synthesized by the two methods. The anodized TiO_2_ nanotubes (Figure 1a,b) are a vertically organized and orderly assembled nanotubular array on the titanium foil surface with an internal diameter of 79 nm, an external diameter of 27 nm, and an approximate length of 2.4 μm. In contrast, the hydrothermal TiO_2_ nanotubes (Figure 1c,d) exhibited a formation of high-density, slender nanotubes with a more random growth orientation on the titanium substrate, each with a diameter of approximately 30 nm. They presented a unique interwoven network microspore structure characterized by deep holes with diameters of less than 1 µm, contributing to their intricate morphology.

Both nanotubes showed a high degree of uniformity in tube diameter, although the organization of the tubes differed markedly between the two methods. The anodized nanotubes were more ordered, while the hydrothermal nanotubes appeared less organized despite their higher density.

**Figure 2:**
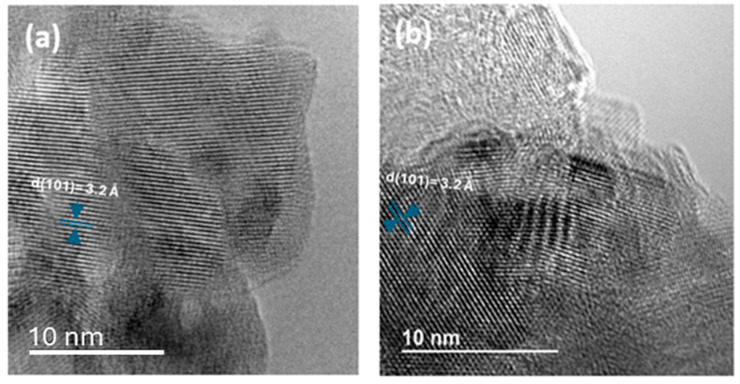
High-resolution TEM images of (**a**) TiO_2_-NTs synthesized with anodization method and (**b**) TiO_2_-NT network synthesized with hydrothermal method.

A high-resolution transmission electron microscopy (TEM) analysis was performed to gain further insights into the structural properties of these nanotubes. The TEM images (Figure 2) highlighted the lattice planes of both types of nanotubes, confirming their high crystallinity. The observed interplanar spacing of d(101) = 3.2 Å across both samples substantiates their highly crystalline nature, indicating well-defined anatase phases. 

These observations suggest that while both synthesis methods yield high-quality TiO_2_ nanotubes with significant crystalline integrity, the morphological characteristics, such as tube organization and density, vary significantly, potentially influencing their respective photocatalytic and photoelectrochemical performances.

### 3.2. Structural Characterization

**Figure 3:**
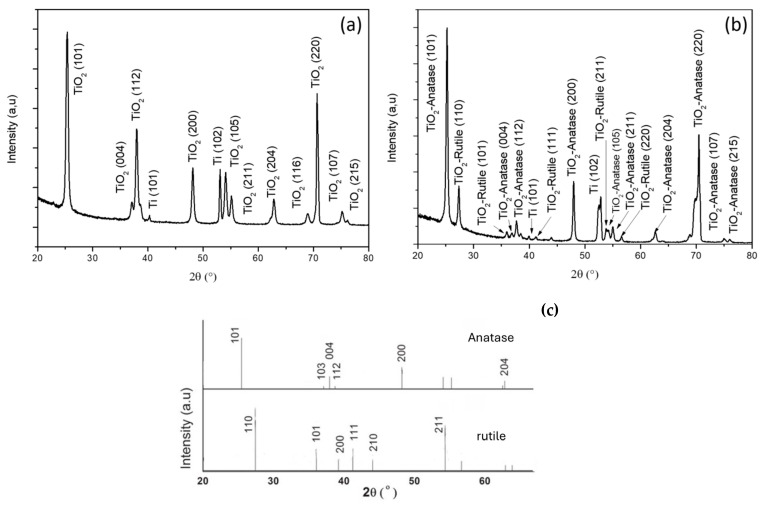
XRD peaks of (**a**) TiO_2_-NTs synthesized with anodization method and (**b**) TiO_2_-NT network synthesized with hydrothermal method; (**c**) reference patterns of anatase and rutile TiO_2_-NTs.

An X-ray diffraction (XRD) analysis was conducted to elucidate the crystalline structures of titanium dioxide nanotubes (TiO_2_-NTs) synthesized via anodization and hydrothermal methods (Figure 3). Both types of TiO_2_-NTs demonstrated a predominant orientation in the anatase phase (101), with respective peaks at 2θ values of 25.4° and 70.5°, as indexed by JCPDS Card 21-1272 [21]. The sharpness of these peaks indicates highly crystalline anatase and titanium in both samples. Additionally, the XRD pattern for the hydrothermally synthesized TiO2-NTs displayed a rutile phase peak at 2θ = 27.5° (JCPDS Card 21-1276 [21]), suggesting a composite phase structure.

The presence of anatase and rutile phases enhances the photocatalytic efficacy of TiO_2_ structures due to the rutile phase’s lower band gap. This facilitates the transfer of photogenerated electrons from the anatase conduction band to the rutile valence band, thereby reducing electron–hole recombination [22]. The proportions of anatase and rutile in the samples were quantified using the integrated intensities of their respective XRD peaks:

(3) $$X\left(\%\right)=\frac{100}{\left(1+1.265\frac{I_{R}}{I_{A}}\right)}$$

where I_A_ and I_R_ represent the intensities of the anatase and rutile peaks at 2θ values of 25.32° and 27.54°, respectively. This calculation yielded an anatase-to-rutile ratio of 74% to 26%. The crystallite size D and microstrain ε were calculated using the Scherrer [23] and Wilson [24] equations, respectively:

(4) $$D=\frac{K\lambda }{\beta \cos\theta }$$

(5) $$\varepsilon =\frac{\beta }{4tan(\theta )}$$

where K is the shape factor, λ is the X-ray wavelength, β is the full width at half maximum of the peak, and θ is the Bragg angle.

The measurements summarized in Table 1 indicate that the anodized TiO_2_-NTs possess a larger crystallite size and lower microstrain compared to the hydrothermal TiO_2_-NT network.

These results suggest a correlation between crystallite sizes and microstrains, with the anodized TiO_2_-NTs exhibiting fewer defects and dislocations than the hydrothermal TiO_2_-NT network. This finding aligns with the observed presence of the rutile phase in the hydrothermal sample, as rutile is generally considered more stable than anatase [25]. A larger crystallite size in the anodized nanotubes could also improve the charge transport efficiency due to fewer grain boundaries for charge carriers to overcome [26].

### 3.3. Optical Characterization

#### 3.3.1. UV–Vis Diffuse Reflectance Spectroscopy (RDS)

**Figure 4:**
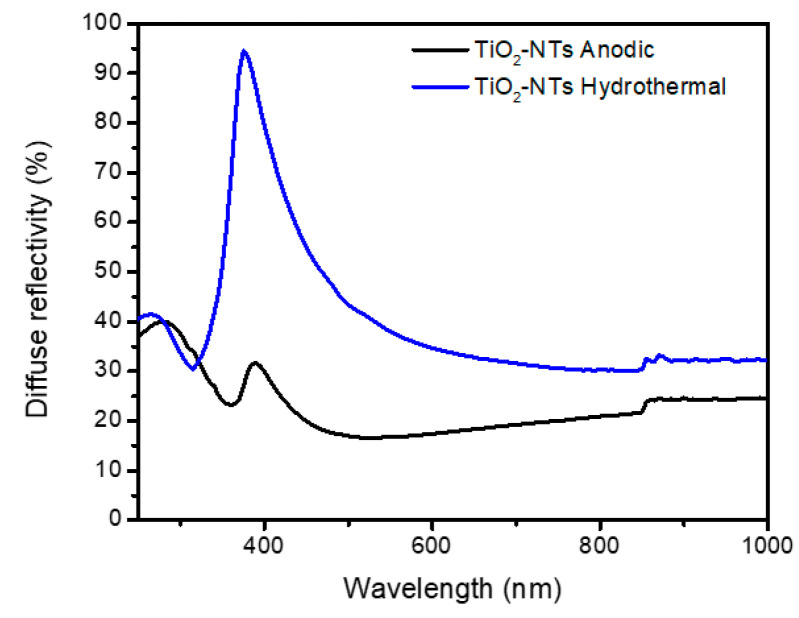
DRS of TiO_2_-NTs synthesized with anodization method and TiO_2_-NT network synthesized with hydrothermal method.

UV–Vis diffuse reflectance spectroscopy (DRS) was utilized to assess the impact of morphological and structural variations on the optical properties of the titanium dioxide nanotubes (TiO_2_-NTs). The DRS results, illustrated in Figure 4, revealed notable differences between the anodized and hydrothermal TiO_2_-NTs. 

The hydrothermally synthesized nanotubes exhibited higher diffuse reflectance, attributed to enhanced light-scattering effects. This increased scattering is primarily due to these nanotubes’ dense and rough morphology, as observed in the SEM images (refer to Figure 1b) [16]. Additionally, these nanotubes displayed a blue color, which might influence the overall reflectance properties. Both types of TiO_2_-NTs showed an absorption peak at approximately 390 nm, deviating from the typical absorption edges seen in other TiO_2_ nanostructures. This anomaly is believed to result from disorder-induced excitation scattering at the molecular level [27]. However, our samples’ specific mechanisms contributing to this behavior warrant further exploration. 

The band gap energy (Eg) of each sample was calculated using the Kubelka–Munk function (F(R)) derived from the diffuse reflectance (R) according to the following equation [28]:

(6) $$F\left(R\right)=\frac{{\left(1-R\right)}^{2}}{2R}=\alpha$$

**Figure 5:**
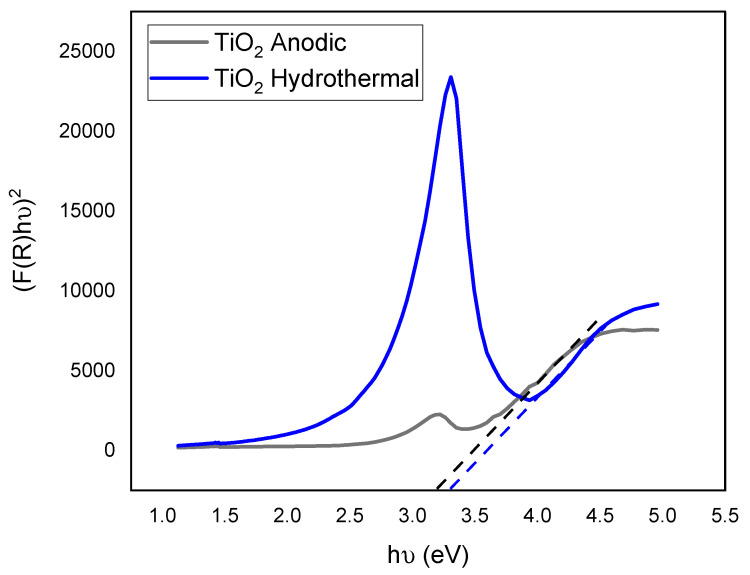
Tauc plot of TiO_2_-NTs synthesized with anodization method and TiO_2_-NT network synthesized with hydrothermal method.

Tauc plots, which graph (F(R) hν)^1/n^ versus photon energy (hν), were constructed (Figure 5) to determine the band gaps through extrapolation. These plots revealed an optical band gap of 3.18 eV for the anodized TiO_2_-NTs and 3.29 eV for the hydrothermally synthesized TiO_2_-NT networks. 

The observed variation in band gaps is primarily attributed to the different crystal phases present in each type of nanotube. Specifically, the difference in band alignment between the rutile and anatase phases, which stems from their distinct ionization potentials and chemical bonding characteristics, likely contributes to the increased band gap observed in the rutile samples [29].

#### 3.3.2. Photoluminescence (PL)

**Figure 6:**
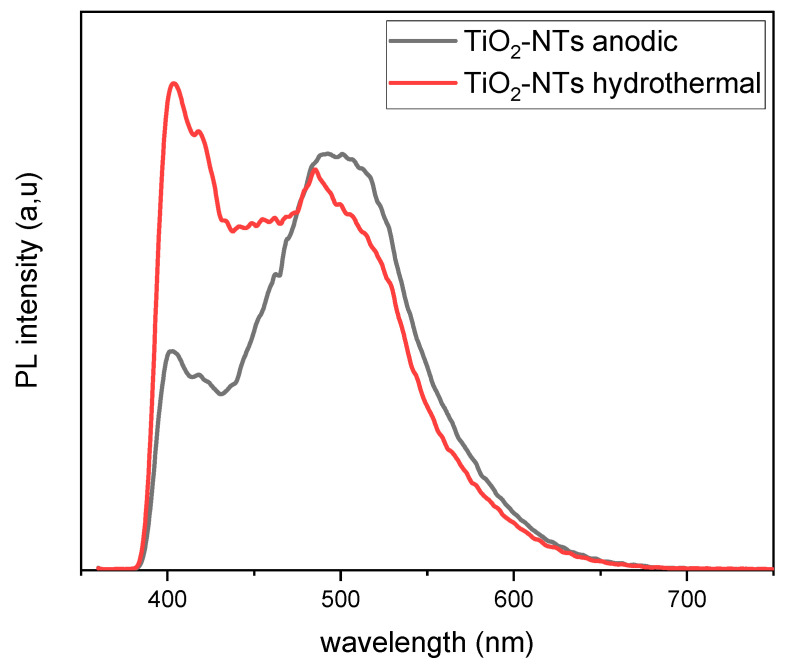
PL spectra of TiO_2_-NTs synthesized with anodization method and TiO_2_-NT network synthesized with hydrothermal method.

Photoluminescence spectroscopy was utilized to discern the effects of morphological and structural differences on the recombination dynamics of photogenerated charge carriers in TiO_2_-NTs. Figure 6 illustrates the PL spectra of TiO_2_ nanotubes synthesized via two disparate methods. A subtle shoulder follows a pronounced peak at 402 nm (3.08 eV) at approximately 418 nm (2.97 eV); these band gap emissions have been linked to the incorporation of carbon impurities during the anodization process [30]. The anodization process facilitates the introduction of impurities such as carbon and fluorine into the TiO_2_ lattice, likely from the electrolytes used in the synthesis [31]. These impurities may also originate from the titanium substrate, as the TiO_2_-NTs in both samples were directly grown on Ti foils [32], leading to additional energy levels within the band gap and consequently narrowing it.

The comparative intensity of these peaks is markedly different for the two samples. The anodized TiO_2_-NTs exhibit a relatively subdued peak, in contrast to the nearly doubled intensity observed for the hydrothermal nanotubes. This difference can be ascribed to the mixed-phase composition of the hydrothermally synthesized TiO_2_-NT network, which consists of both anatase and rutile phases; the latter’s higher weight percentage (26%) is known to accelerate recombination rates [33]. Additionally, interference from the granulated TiO_2_ film, which potentially forms between the Ti foil and hydrothermally grown nanotubes, might create recombination sites for electron–hole pairs.

The cluster of peaks around 484 nm is generally associated with oxygen vacancies in the TiO_2_ structure, acting as electron traps. The diminished emission intensity for these peaks in the hydrothermally synthesized nanotubes implies a lower concentration of oxygen vacancies, likely due to the stabilizing presence of the rutile phase, as rutile is more stable than anatase. Conversely, the pronounced peaks in the anodized sample could be due to its superior tubular morphology, which enhances charge separation and reduces recombination at oxygen vacancy sites [34].

Thus, the PL spectra corroborate a decreased band gap for the anodized TiO_2_-NTs, as observed in Figure 5, underscoring the complex interplay between morphology, structural phases, and the electronic properties of TiO_2_ nanotubes [34].

#### 3.3.3. Hydrophilicity of TiO_2_-NTs

Hydrophilicity, a measure of a material’s affinity for water, is a crucial characteristic of TiO_2_ nanotubes (TiO_2_-NTs) that significantly impacts their photocatalytic efficiency [35]. During photocatalysis under UV irradiation, electron–hole pairs (e^−^/h^+^) are generated within the TiO_2_-NTs. These pairs can recombine on the surface, reducing their photocatalytic activity, or react with adsorbed water molecules and surface hydroxyl groups. These reactions lead to the formation of reactive oxygen species (ROS), which are highly effective oxidizing agents responsible for the degradation of pollutants. Enhanced water adsorption on a more hydrophilic surface facilitates the generation of ROS, ultimately leading to superior photocatalytic performance.

**Figure 7:**
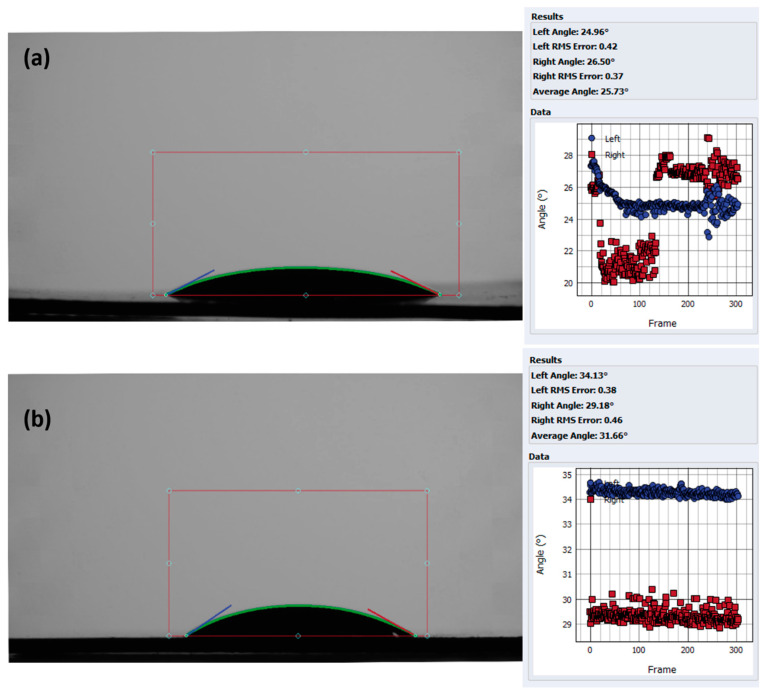
Contact angle of (**a**) TiO_2_-NTs synthesized with anodization method and (**b**) TiO_2_-NT network synthesized with hydrothermal method.

Figure 7 depicts the contact angle measurements for both types of TiO_2_-NTs. As expected, the anodized TiO_2_-NTs (Figure 7a) exhibit a notably lower average contact angle (25.73° ± 0.39°) compared to the hydrothermally synthesized TiO_2_-NTs (Figure 7b), with an average contact angle of 31.66° ± 0.42°. This difference of 5.93° indicates a significantly higher degree of hydrophilicity for the anodized samples. Consequently, we can expect more adsorbed water molecules on the surface of the anodized TiO_2_-NTs, potentially leading to enhanced ROS generation and improved photocatalytic activity [35].

#### 3.3.4. Photoelectrochemical Characterization

The photoelectrochemical behavior of the TiO_2_-NTs was evaluated using a standard three-electrode configuration under irradiation from a Xenon lamp. Both samples, the anodized TiO_2_-NTs and the hydrothermally synthesized TiO_2_-NT network, have the same dimensions. Linear sweep voltammetry was conducted in dark and light conditions using 1M NaOH as the supporting electrolyte at a scan rate of 5 mV/s. The photoelectrochemical mechanism can be detailed as follows: when using an n-type semiconductor photoelectrode, photoexcited electrons move via the back contact and external circuit to the counter electrode, where they decrease water, while photogenerated holes migrate to the photoelectrode/electrolyte interface and perform water oxidation. The n-type semiconductor functions as the photoanode in this method, and to enable effective oxygen evolution, its valence band level needs to be more positive than the H_2_O/O_2_ potential (+1.23 V vs. RHE). However, since the potential shortage can be made up by introducing an external voltage, the potential of the photoexcited electrons at the counter electrode can be less negative than the H^+^/H_2_ potential (0 V vs. RHE) depending on the photoanode’s Fermi level.

**Figure 8:**
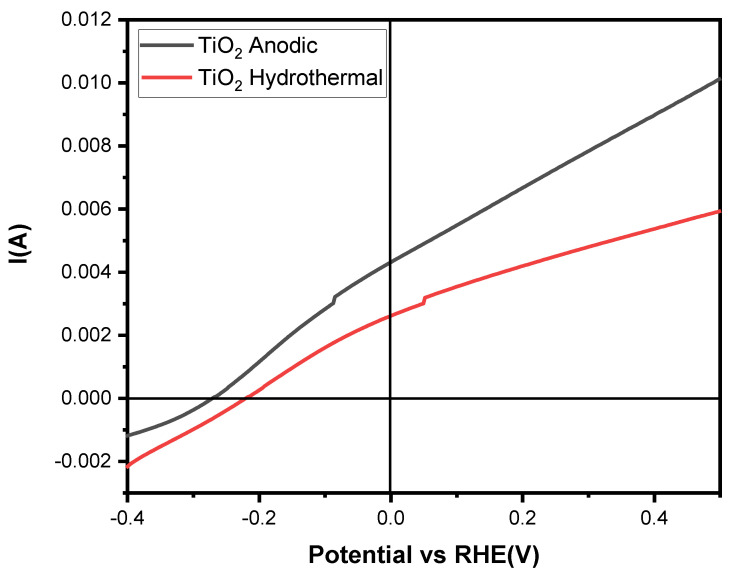
LSV linear voltammograms of TiO_2_-NTs synthesized by the anodization and hydrothermal methods.

Figure 8 depicts the linear voltammograms, showing a higher current for the anodized TiO_2_-NTs than the hydrothermally synthesized TiO_2_-NTs. This difference can primarily be attributed to the higher photocatalytic activity and better carrier transport properties of anatase, even though the hydrothermal nanotubes, which consist of a biphasic anatase and rutile structure, were expected to aid in charge separation. However, the 26% rutile weight percentage determined by the XRD analysis exceeds the optimal value for enhancing photocurrent response [36].

**Figure 9:**
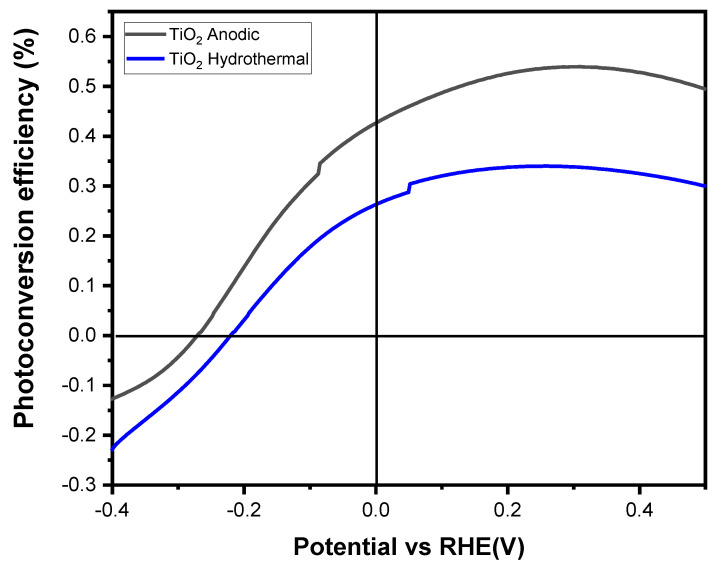
Variation in the photoconversion rate vs. Ag/AgCl potential for TiO_2_-NTs synthesized by anodization and hydrothermal methods.

Figure 9 illustrates the photoconversion efficiency for each sample; the anodized nanotubes demonstrate a maximum photoconversion rate of 0.54% at a potential of 0.28 V vs. Ag/AgCl, which is 0.2% higher than that of the hydrothermally synthesized TiO_2_ nanotubes. According to S. Ozkan et al., TiO_2_-NTs with a rutile-to-anatase weight ratio of 7.4% to 92.6% exhibit a higher photoresponse than those with a 27.4% rutile to 72.6% anatase ratio. This decline in photoconversion efficiency with increased rutile content is attributed to the slower electron mobility associated with rutile, which reduces the number of charge carriers reaching the electrode, thus decreasing the photoresponse [37].

Additionally, the anodized TiO_2_-NT electrodes exhibited an improved photocurrent when irradiated compared to their hydrothermal counterparts in dark and light conditions. This achievement is attributed to the enhanced mobility of charge carriers under illumination. The hydrothermal nanotube structure’s lower crystallinity and higher defect density create more recombination centers, reducing the photocurrent intensity. Microstrain calculations show that the hydrothermal sample exhibited a significantly higher microstrain of 0.61%, indicating a greater prevalence of point and structural defects, which lead to slower charge transport and increased recombination [38,39]. These findings are consistent with the PL intensity results, where the hydrothermal TiO_2_-NTs demonstrated higher intensity, indicative of a higher recombination rate and reduced generation of e^−^/h^+^ pairs. 

#### 3.3.5. Photocatalytic Characterization

Photocatalytic performance is intrinsically linked to the morphology and structure of nanophotocatalysts. These factors influence the generation of electron–hole pairs (e^−^/h^+^) and, subsequently, the production of reactive oxygen species (ROS), primarily superoxide radicals (O_2_^•−^) and hydroxyl radicals (^•^OH). These dynamics are outlined by the following reactions [40]:

TiO_2_ + hν→TiO_2_ (e^−^ CB + h^+^ VB) (7)

e^−^CB + O_2_ → O_2_^•−^ (8)

O_2_^•−^ +H_2_O→^•^OH + ^−^OH + O_2_ (9)

h^+^ VB + H_2_O→^•^OH (10)

(^•^OH, O_2_^•−^) + Dye → Degradation products (11)

**Figure 10:**
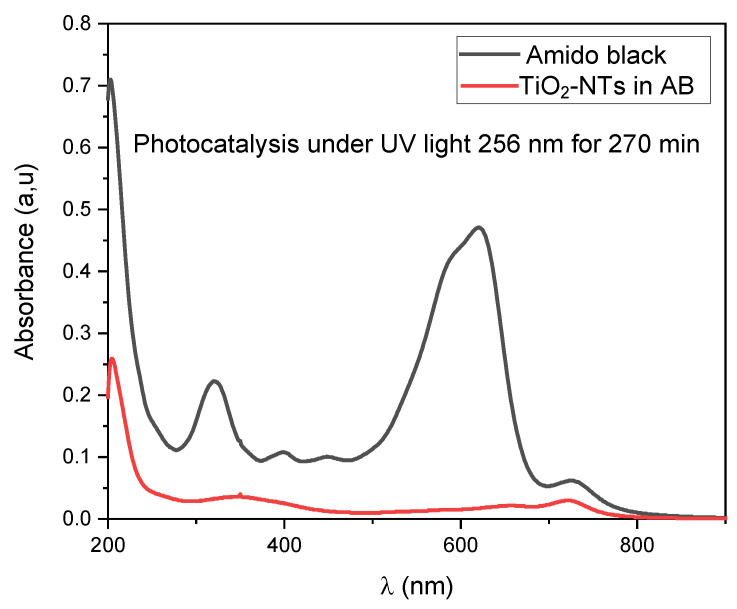
The absorbance spectra of amido black with irradiation time with and without TiO_2_-NTs.

For the amido black staining degradation, an experiment was conducted as is shown in Figure 10 below. After 270 min of photocatalysis under 256 nm UV light, the amido black did not degrade, and only in the presence of TiO_2_-NTs the peaks were significantly decreased. 

**Figure 11:**
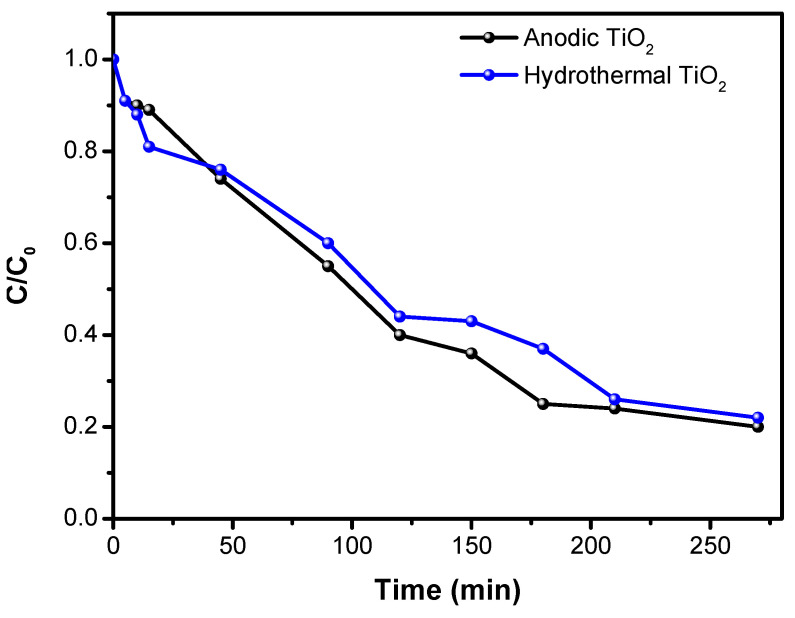
Variation in C/C_0_ of amido black with irradiation time for TiO_2_-NTs synthesized with anodization method and TiO_2_-NT network synthesized with hydrothermal method.

Figure 11 illustrates how nanotubular characteristics like self-organization, tube length and diameter, and electronic and ionic properties can significantly impact photocatalytic performance. The morphological and structural differences between TiO_2_-NTs synthesized via the hydrothermal method and those by the anodization method directly affect degradation efficiency. The hydrothermally synthesized TiO_2_-NTs show a slightly lower efficiency (78%) than the anodized TiO_2_-NTs (80%).

The XRD results indicate that anodic TiO_2_-NTs exhibit higher intensity for the (004) facet than hydrothermally synthesized TiO_2_-NTs [41]. This finding suggests the superior crystallinity of this facet, known for its high photocatalytic activity, which correlates with the observed performance difference. Additionally, anodic nanotubes display better light absorption and lower PL intensity, both favorable for generating more electron–hole pairs and, consequently, more ROS.

The samples’ hydrophilicity can also influence the adsorption of organic pollutants, thus affecting photodegradation efficiency [42]. According to the hydrophilic tests, the anodic nanotubes showed better wettability than the hydrothermal nanotubes, with a significant 5.93° difference in the contact angle, enhancing their photocatalytic performances.

**Figure 12:**
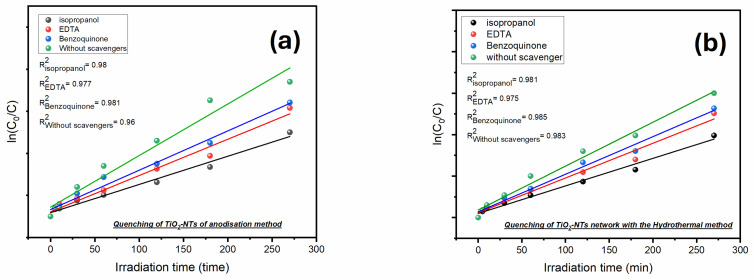
The logarithm plots of (**a**) TiO_2_-NTs synthesized with anodization method and (**b**) TiO_2_-NT network synthesized with hydrothermal method.

To further dissect the roles of various ROS in photodegradation, specific scavengers were added to the dye solution: benzoquinone (quenches superoxide radicals), EDTA (traps holes), and isopropanol (neutralizes hydroxyl radicals). Figure 12 illustrates that adding these scavengers significantly impedes the degradation of amido black, with a marked reduction in the rate for each added scavenger. The degradation kinetics were analyzed using the following equation [43]:

(12) $$\ln\left(\frac{C_{0}}{C_{t}}\right)=Kt$$

*C*_0_ and *C_t_* are the dye concentrations at the initial time and time *t*, respectively, and *K* is the rate constant. The slopes derived from Figure 12 quantify the photodegradation rate ‘*k*’ showcasing each scavenger’s impact on each sample’s performance.

**Figure 13:**
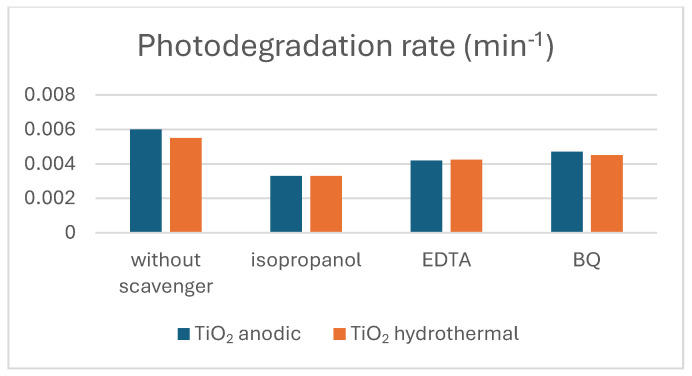
Evaluation of photodegradation rate of TiO_2_-NTs synthesized with anodization method and TiO_2_-NT network synthesized with hydrothermal method with and without scavengers.

It is observed that the addition of isopropanol significantly reduces the photodegradation rate to half of the rate of the sample without scavengers, as shown in Figure 13. The assimilation of benzoquinone (BQ) also reduces the rate constant for photocatalysis, but to a lesser extent than isopropanol. This indicates that hydroxyl radicals and superoxide radicals are the primary reactive oxygen species (ROS) involved in the photocatalytic performance of both samples. Additionally, the incorporation of EDTA slightly decreases the rate constant, indicating that holes play a moderate role in the photodegradation mechanism but less than the primary ROS. 

## 4. Conclusions

This study has elucidated several critical insights from a detailed comparison between anodized TiO_2_-NTs and hydrothermally synthesized TiO_2_-NT networks, focusing on their morphological, structural, photoelectrochemical, and photocatalytic properties. The X-ray diffraction analysis highlighted that hydrothermally synthesized TiO_2_-NTs contain a significant rutile phase of 26% by weight, indicating a higher prevalence of structural defects than the anodized samples. Additionally, variations in band gap energies, as revealed by UV–Vis spectroscopy, pointed to differing levels of oxygen vacancies between the samples, a finding supported by photoluminescence spectra.

The empirical assessments demonstrated that anodized TiO_2_-NTs, with their uniform single-phase structure, exhibit superior photocatalytic and photoelectrochemical performances. Notably, these nanotubes achieved a photoconversion efficiency of 0.54% at a potential of 0.28 V vs. Ag/AgCl. Their enhanced activity is attributed to their well-defined structure, precise dimensions, high organizational integrity, and hydrophilic surfaces, which facilitate effective interactions with water molecules. The photodegradation rate of the anodized TiO_2_-NTs was recorded at 0.006 min^−1^, significantly surpassing the hydrothermal TiO_2_-NTs, which showed a rate constant of 0.005 min^−1^.

The comparative analysis between the anodized and hydrothermally synthesized TiO_2_-NTs highlights the advantages of using anodized single-phase nanotubes for photocatalytic and photoelectrochemical applications. The meticulous control over structural properties in the anodized TiO_2_-NTs, such as dimensionality, organization, and surface characteristics, contributes significantly to their superior performance. These findings not only underline the critical role of fabrication techniques in optimizing the functional properties of TiO_2_ nanotubes but also provide valuable insights for their application in wastewater treatment technologies utilizing photocatalysis for efficient pollutant degradation.

## Figures and Tables

**Table 1:** Crystallite size measurements and microstrain of TiO_2_-NTs synthesized with anodization and hydrothermal methods.

	TiO_2_ Phase	Crystallite Size	Microstrain
**Hydrothermal TiO_2_-NT network**	TiO_2_(101)-anatase	23.3 nm	6.8 × 10^−3^
	TiO_2_(110)-rutile	22.7 nm	6.4 × 10^−3^
**Anodized TiO_2_-NTs**	TiO_2_(101)-anatase	30.5 nm	5.1 × 10^−3^

## Data Availability

The original contributions presented in the study are included in the article, further inquiries can be directed to the corresponding author.
